# Interleukin 35 Synovial Fluid Levels Are Associated with Disease Activity of Rheumatoid Arthritis

**DOI:** 10.1371/journal.pone.0132674

**Published:** 2015-07-23

**Authors:** Ladislav Šenolt, Barbora Šumová, Romana Jandová, Hana Hulejová, Heřman Mann, Karel Pavelka, Jiří Vencovský, Mária Filková

**Affiliations:** 1 Institute of Rheumatology, Prague, Czech Republic; 2 Department of Rheumatology, 1^st^ Faculty of Medicine, Charles University in Prague, Prague, Czech Republic; SERGAS (Servizo Galego de Saude) and IDIS (Instituto de Investigación Sanitaria de Santiago), Santiago de Compostela, SPAIN

## Abstract

**Objectives:**

To study the association of systemic and local interleukin-35 (IL-35) levels in rheumatoid arthritis.

**Methods:**

37 patients with treatment naïve early RA, 49 with established RA and 29 control patients with osteoarthritis (OA) were studied. Serum and paired synovial fluid samples were analysed for IL-35. Disease activity of RA patients was assessed according to the 28-Joint Count Disease Activity Score (DAS28).

**Results:**

The levels of serum IL-35 were significantly higher in patients with treatment naïve early RA compared to those with established disease and control OA subjects. In addition, serum levels of IL-35 significantly decreased 12 weeks after initiation of glucocorticoids and conventional synthetic disease modifying antirheumatic drugs in patients with treatment naïve early RA. Synovial fluid IL-35 levels were significantly higher in RA compared to OA patients, were significantly elevated compared to serum counterparts and correlated with synovial fluid leukocyte count (r=0.412; p<0.01), serum CRP levels (r=0.362; p<0.05) and DAS28 (r=0.430, p<0.01).

**Conclusion:**

This is the first study showing elevated circulating levels of IL-35 in treatment naïve early RA, its significant decrease after treatment initiation and positive association between increased synovial fluid IL-35 and disease activity in patients with long-lasting RA.

## Introduction

Rheumatoid arthritis (RA) is a chronic autoimmune disease characterized by persistent synovial and systemic inflammation causing joint damage, disability, increased morbidity and mortality [1]. A large number of cytokines produced by activated immune cells and synovial fibroblasts in the rheumatoid joints play a fundamental role in the process of RA [2, 3]. Despite new insights into the pathogenesis of RA, novel therapeutic strategies and profound efficacy of biologics in RA, the cause of the disease, long lasting disease remission or even complete cure remain still elusive [4–6]. Therefore, identification of new cytokines offers promise for comprehensive understanding to the pathogenesis of RA and may potentially point to novel targets for future therapies.

Interleukin-35 (IL-35), a heterodimeric cytokine composed of the subunits EBI3 and p35, was recently identified as a new member of the IL-12 cytokine family [7]. Although IL-12, a pro-inflammatory cytokine, plays a critical role in the differentiation of Th1 cells, IL-35 has been described as an anti-inflammatory cytokine, at least in mice [8, 9)]. IL-35 effectively attenuated established collagen-induced arthritis and experimental colitis in mice [8, 9]. This immunosuppressive potential of IL-35 in mouse might be partially explained by attenuated function of Th1 and Th17 cells [8, 9]. On the contrary, IL-35 failed to prevent development of Lyme arthritis in mice but instead enhanced the inflammatory response in this model [10]. Further, IL-35 gene therapy exacerbated experimental arthritis and promoted chronic inflammation [11]. In line with this, we have recently shown up-regulation of IL-35 in RA synovial tissue and its pro-inflammatory properties in humans [12].

Several reports have demonstrated an association between circulating IL-12 family members and disease activity of RA [13–15]. Therefore the aim of the present study was to evaluate serum and synovial fluid levels of IL-35 in patients with early and established RA and to determine its potential association with disease activity.

## Methods

### Patients

Altogether, 49 patients with established RA (disease duration 15.1 ±10.7 years) fulfilling the American College of Rheumatology (ACR) criteria for the classification of RA [16], 37 treatment naïve early RA patients fulfilling the ACR/EULAR 2010 classification criteria with symptom duration of less than six months [17] and 29 control patients with knee OA were enrolled into this study. In patients with early RA, treatment with glucocorticoids (n = 31; mean daily dose at month three was 5 mg; range 1.25–15.0 mg of prednisone or equivalent per day), methotrexate (n = 27, mean weekly dose at month three was 15 mg; range 10–20 mg), sulphasalazine (n = 6; mean daily dose was 2 g) and leflunomide (n = 6; mean daily dose was 20 mg) was initiated and given throughout the study. More individuals from the group of early RA patients were given glucocorticoids compared to long-term treated RA patients (83.7 vs. 63.3%). In addition, long-term treated RA patients received either conventional synthetic disease modifying antirheumatic drugs (cs-DMARDs) or biologic therapy (as monotherapy or in combination with cs-DMARDs), while none of the early RA patients received biologic therapy.

Disease activity of RA patients was assessed according to the 28-Joint Count Disease Activity Score (DAS28) using the number of swollen and tender joints, erythrocyte sedimentation rate (ESR) and patient responses using the global visual analogue scale (VAS). The patient characteristics are given in Table 1. The study was approved by the Ethics Committee of the Institute of Rheumatology and written informed consents were obtained from all patients prior to initiation of the study.

**Table 1:** Demographic Characteristics of Patients with Treatment Naïve Early and Established Rheumatoid Arthritis and Control Individuals with Osteoarthritis.

	ERA, wk 0 (n = 37)	ERA, wk 12 (n = 37)	RA (n = 49)	OA (n = 29)
**Demographic characteristic**				
Age (years)	48.0 ± 15.1	48.0 ± 15.1	55.9 ± 13.3*	64.8 ± 10.5^±^ ^§^
Sex (% female)	73.0	73.0	73.5	58.6
BMI (kg/m^2^)	24.6 ± 3.8	NA	25.3± 3.8	27.6 ± 3.8^≠^ ^§^
**Disease activity**				
DAS28-ESR (score)	5.1 ± 1.4	2.6 ± 1.4***	4.6 ± 1.5	-
CRP (mg/l)	13.4 ± 17.0	4.3 ± 6.1***	25.7 ± 32.6	3.6 ± 5.2^ǂ^
**Medication**				
glucocorticoids (%)	-	83.7	63.3	
csDMARDs (%)	-	94.6	83.7	-
bDMARDs (%)	-	0	22.4	-
**Autoantibody positivity**				
RFs (%)	54	NA	62	-
Anti-CCP (%)	51	NA	59	-

Values are the mean ± SD, unless otherwise stated. BMI, body mass index; DAS, disease activity score; ESR, erythrocyte sedimentation rate; CRP, C-reactive protein; csDMARDs, conventional synthetic disease modifying antirheumatic drugs; bDMARDs, biologic disease modifying antirheumatic drugs; RFs, rheumatoid factors; anti-CCP, anti-cyclic citrullinated peptide; NA, not applicable. Types of bDMARDs: anti-TNFα (etanercept 3x, adalimumab 2x and golimumab 1x), anti-CD20 (rituximab 2x), anti-IL6R (tocilizumab 2x) and anti-IL17 (secukinumab 1x, open phase clinical trial).

*p<0.05 for pairwise comparisons with ERA;

^±^p<0.001 for pairwise comparisons with ERA;

^§^p<0.01 for pairwise comparisons with RA;

^≠^p<0.01 for pairwise comparisons with ERA;

***p<0.001 for pairwise comparisons with ERA wk 0;

^ǂ^p<0.001 for pairwise comparisons with ERA and RA

### Laboratory Analysis

The blood samples were collected within five days after arthrocentesis from patients with established RA and knee OA who had the procedure done from therapeutic reasons. Paired blood and synovial fluid samples were immediately centrifuged and were stored at -80°C until analysed. Before analysis, the synovial fluids were incubated with Hylase Dessau for 30 min at 37°C. In addition, serum samples were taken from treatment naïve early RA patients before and 12 weeks after initiation of treatment.

Serum and synovial fluid levels of IL-35 were measured by ELISA according to manufacturer’s protocol (USCN Life Science, Wuhan, China). Absorbance was detected using the Sunrise ELISA reader (Tecan, Salzburg, Austria) with 450 nm as the primary wavelength. Immuno-turbidimetric technique was used to measure the CRP levels using an Olympus biochemical analyser (model AU 400, Japan). The levels of anti-cyclic citrullinated peptide (anti-CCP) antibodies and IgM rheumatoid factors (IgM-RF) in serum were determined by an ELISA (Test Line s.r.o., Czech Republic).

### Statistical Analysis

A Kolmogorov-Smirnov test of normality was performed for all variables. The levels of IL-35 were described as median (range) and the levels of DAS28 were expressed as mean (SD). For comparisons between groups, the unpaired t-test or Mann-Whitney test were used. Wilcoxon matched pairs test was used to compare paired samples. Pearson’s product-moment correlations and Spearman’s rank correlations were used in cases of normal and non-normal distribution, respectively. P values below 0.05 were considered to be statistically significant. Correlation coefficient 0.1–0.3 was considered weak, 0.3–0.5 was considered moderate and 0.5–1.0 was a strong correlation. Statistical analyses were performed using SPSS 17.

## Results

### Serum Levels of IL-35 Are Higher in Patients with RA

**Fig 1:**
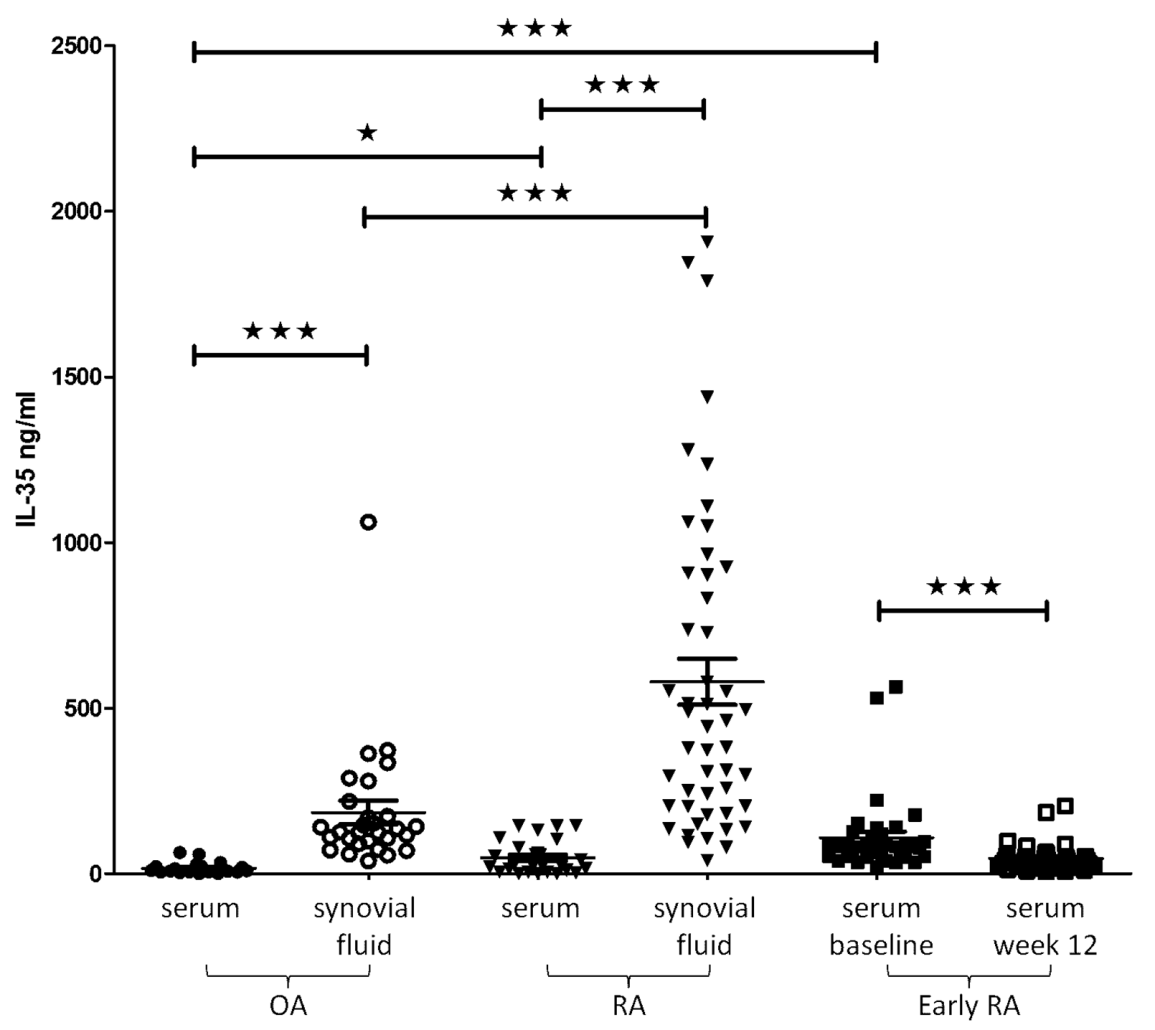
Circulating and synovial fluid levels of IL-35 in patients with established and early rheumatoid arthritis and subjects with osteoarthritis. The introduction of treatment in early RA resulted in significantly decreased levels of IL-35 after 12 weeks of therapy. OA, osteoarthritis; RA, rheumatoid arthritis.

The levels of serum IL-35 were significantly higher in patients with treatment naïve early RA at baseline compared to control patients with OA as well as to those with established RA (81.6 [20.7–564.4] vs 10.4 [0.6–64.1] vs 22.8 [1.2–145.5] pg/ml; p<0.001 for both comparisons) and significantly decreased after treatment initiation (to 36.5 [5.0–204.8] pg/ml; p<0.001) (Fig 1). The levels of IL-35 in early RA patients following treatment were comparable to that in established RA patients, but did not significantly differ from that in control OA subjects (Fig 1). However, the levels of serum IL-35 were significantly higher in patients with established RA compared to control OA subjects (p<0.05).

### IL-35 in Synovial Fluid Is Associated with RA Disease Activity

Since RA patients had elevated serum levels of IL-35, we further investigated whether IL-35 levels could be associated with RA disease activity. However, no significant associations were found between serum IL-35 levels, CRP or DAS28 either in early or in established RA patients. Decrease in IL-35 levels after 12 weeks of treatment also did not correlate with clinical improvement over time in early RA patients (data not shown). Serum levels of IL-35 were not affected by age, sex and IgM-RF or anti-CCP autoantibodies.

**Fig 2:**
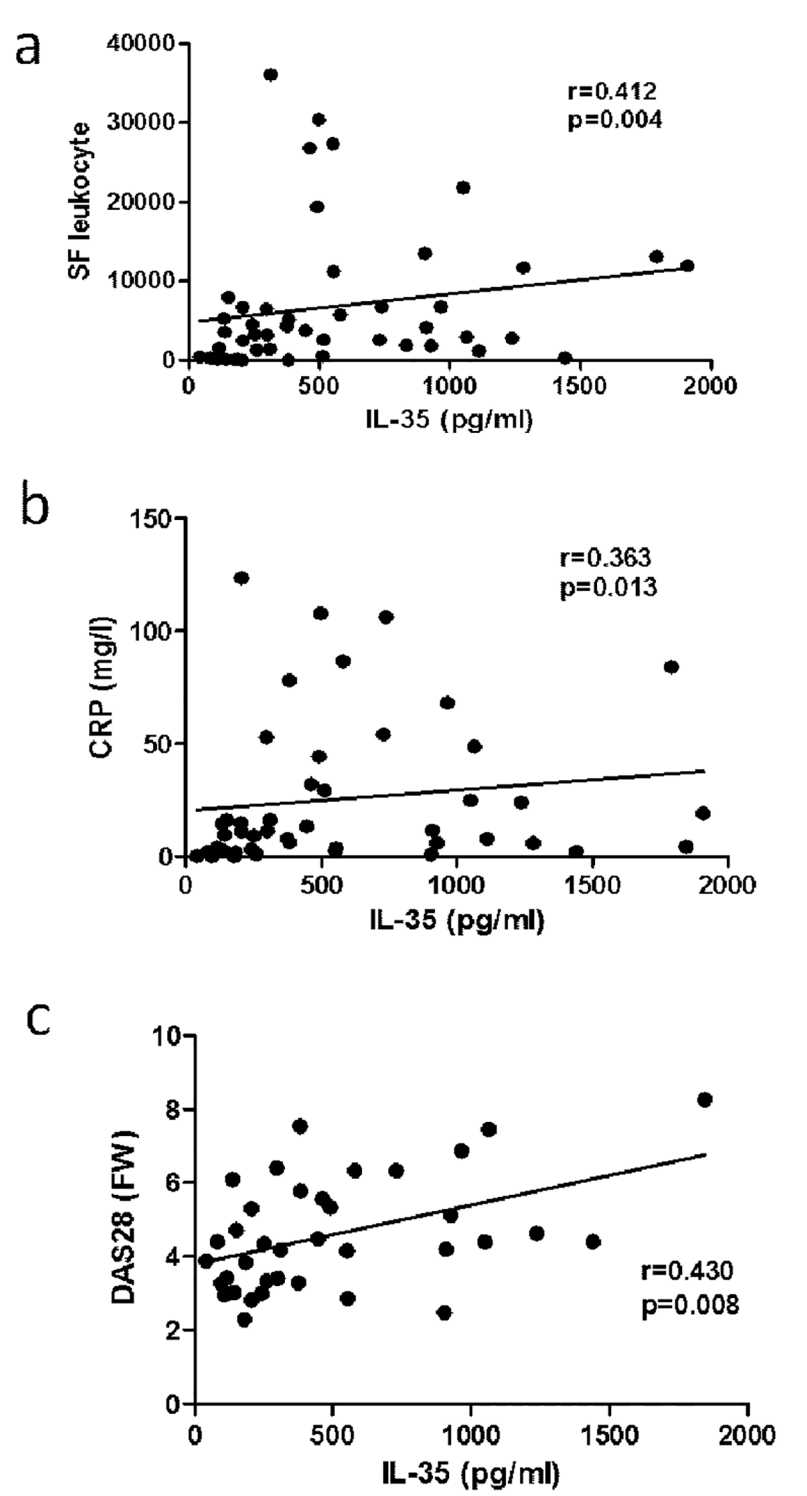
Associations of IL-35 levels in synovial fluid with synovial fluid leukocyte count (a), serum CRP levels (b) and DAS28-ESR (c). CRP, C-reactive protein; DAS28, 28 joint disease activity score calculated with ESR, erythrocyte sedimentation rate.

Next, we compared the local levels of IL-35 between established RA patients and control OA subjects. We found that synovial fluid IL-35 levels were significantly higher in RA patients compared to OA subjects (445.0 [40.7–1908.0] vs. 125.5 [39.1–1062.0]; p = <0.001) (Fig 1). Interestingly, there was a moderate correlation between synovial fluid IL-35 levels and synovial fluid leukocyte count (r = 0.412; p<0.01), disease activity assessed by CRP (r = 0.362; p<0.05) and DAS28 (r = 0.430, p<0.01) (Fig 2). In contrast to serum, synovial fluid IL-35 levels correlated with anti-CCP levels (r = 0.444; p = <0.01), but not with IgM-RF (r = 0.110, p = 0.479). When adjusted to anti-CCP levels, the abovementioned associations remained unchanged.

### Serum Levels of IL-35 Are Higher in Patients with RA

#### IL-35 is up-regulated at local sites of inflammation

Our recent finding demonstrated up-regulation of IL-35 in RA synovial tissue [12]. Therefore, we sought to analyse differences between local and circulating IL-35. We found that synovial fluid IL-35 levels were significantly higher compared to that in serum counterparts in RA (445.0 [40.7–1908.0] vs. 22.8 [1.2–145.5] pg/ml; p<0.001) as well as in OA subjects (125.5 [39.1–1062.0] vs. 10.35 [0.600–64.10] pg/ml; p<0.001) (Fig 1). However, there was no association between serum and synovial fluid IL-35 levels in either RA patients or OA subjects.

## Discussion

This is the first study showing elevated serum levels of IL-35 in patients with RA and their significant decrease in treatment naïve early RA following initiation of therapy. Furthermore, higher local, but not systemic, production of IL-35 significantly correlated with higher disease activity supporting a potential role of IL-35 in the pathogenesis of RA.

Circulating members of IL-12 family have been found to correlate with disease activity of RA [13–15]. Increased systemic levels of IL-12, IL-27 and a trend towards higher IL-23 levels were shown in patients with RA compared to control individuals [13–15]. Furthermore, RA patients with detectable IL-12 levels had higher disease activity than those with undetectable IL-12 levels [13]. In addition, levels of IL-23 correlated strongly with RA disease activity [14]. IL-35 belongs to the IL-12 family of cytokines and consistently with abovementioned data; we found that serum IL-35 levels were elevated in RA, particularly in early treatment naïve phase of the disease. Furthermore, we observed that initiation of conventional therapy contributed to significant decrease of IL-35 levels, as the levels of serum IL-35 after commencing treatment in early RA patients were comparable to that in established RA patients on long-term therapy. To support this data, we also found that IL-35 serum levels were increased in patients with systemic sclerosis, particularly in those with early inflammatory stages of the disease, compared to healthy controls (Tomcik, et al., Rheumatology, accepted fro publication). In addition, circulating IL-35 is higher in patients with different types of cancer; its levels correlate with clinical stages of the tumour [18–20] and significantly decrease after the surgical resection [20]. However, in patients with multiple sclerosis, serum levels of IL-35 do not differ between untreated patients and control subjects [21]. Interestingly, in patients with systemic lupus erythematosus [22] and inflammatory bowel diseases [23], the levels of serum IL-35 are even lower compared to healthy subjects and inversely associated with the disease activity. In contrast to these data, we did not observe any association between serum IL-35 levels and RA disease activity either in early or in established disease. Furthermore, we did not observe any influence of concomitant biologics on the levels of IL-35 either in serum or synovial fluid. It would suggest that local production of IL-35 might reflect disease activity rather than type of treatment and might be more important to pathogenic mechanisms of RA. Further studies are therefore needed to elucidate any role of circulating IL-35 in monitoring disease activity of the processes associated with inflammation, autoimmunity and cancer.

Recently, we demonstrated significant up-regulation of IL-35 in RA synovial tissue compared to that in OA and psoriatic arthritis [12]. We showed that both IL-35 subunits are expressed in synovial fibroblasts and, importantly, in several types of immune cells. This expression was further triggered by TNFα [12]. In line with this, our current data show significant elevation of the local IL-35 in established RA and association between IL-35 concentration and total leukocyte count in synovial fluid. Furthermore, IL-35 in synovial fluid significantly correlated with disease activity, which, together with our recent finding that IL-35 dose-dependently induces secretion of pro-inflammatory cytokines in mononuclear cells [12], supports a possible role of IL-35 in RA pathogenesis. We hypothesize that local upregulation of IL-35 in RA synovial tissue and synovial fluid is a result of activation of both synovial fibroblasts and immune cells, and thus reflects disease activity.

In conclusion, we demonstrate here elevated circulating IL-35 in RA, particularly at early phase of the disease, its significant decrease after treatment initiation and a positive association between synovial fluid IL-35 and RA disease activity. These data further support an involvement of IL-35 in the pathogenesis of RA.
